# Iterative class discovery and feature selection using Minimal Spanning Trees

**DOI:** 10.1186/1471-2105-5-126

**Published:** 2004-09-08

**Authors:** Sudhir Varma, Richard Simon

**Affiliations:** 1 Biometric Research Branch, National Cancer Institute, Rockville, USA

## Abstract

**Background:**

Clustering is one of the most commonly used methods for discovering hidden structure in microarray gene expression data. Most current methods for clustering samples are based on distance metrics utilizing all genes. This has the effect of obscuring clustering in samples that may be evident only when looking at a subset of genes, because noise from irrelevant genes dominates the signal from the relevant genes in the distance calculation.

**Results:**

We describe an algorithm for automatically detecting clusters of samples that are discernable only in a subset of genes. We use iteration between Minimal Spanning Tree based clustering and feature selection to remove noise genes in a step-wise manner while simultaneously sharpening the clustering.

Evaluation of this algorithm on synthetic data shows that it resolves planted clusters with high accuracy in spite of noise and the presence of other clusters. It also shows a low probability of detecting spurious clusters. Testing the algorithm on some well known micro-array data-sets reveals known biological classes as well as novel clusters.

**Conclusions:**

The iterative clustering method offers considerable improvement over clustering in all genes. This method can be used to discover partitions and their biological significance can be determined by comparing with clinical correlates and gene annotations. The MATLAB^© ^programs for the iterative clustering algorithm are available from

## Background

**Figure 1:**
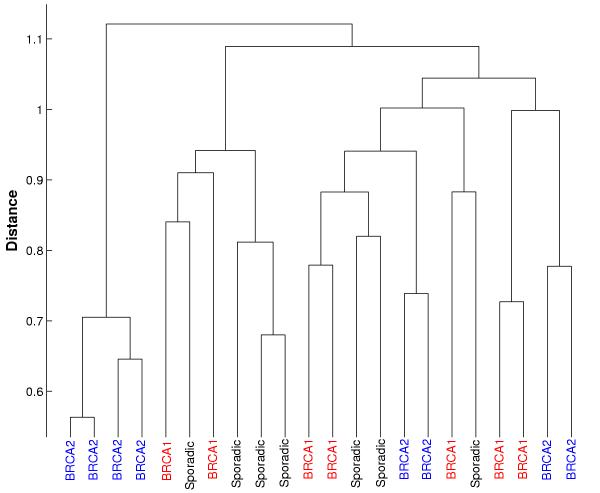
**Hierarchical clustering of BRCA data using all genes. **Hierarchical clustering of BRCA data using centered correlation with average linkage. Inclusion of all genes in the clustering swamps out the differences between samples with BRCA1 and BRCA2 mutation.

Clustering is one of the most common methods for discovering hidden structure in micro-array gene expression data. Clustering of samples has been used to discover new disease taxonomies [1-3]. Cluster analysis is often performed with hierarchical [4], K-means [5] or Self-Organizing Map [6] algorithms, using the entire set of genes as the basis for calculating pair-wise distances between samples. This gives equal weights to the expression of all genes and may be effective in cases where there is a large difference between subsets of samples (*e.g. *comparing samples of normal and cancerous tissues). Many diseases, though, are characterized by small numbers of genes that differentiate between different disease states. Giving equal weight to relevant and irrelevant genes will obscure this difference. Figure 1 shows an example, where clustering in all genes masks the biological differences between samples with BRCA1 and BRCA2 mutation (data from Hedenfalk *et al *[7])

In this article we propose an iterative algorithm, where we initially do a clustering using all the genes. This clustering (which gives a binary partition of the samples) is used to select genes that differentiate between the two clusters. The clustering is done again, but this time, only in the set of genes that was selected in the previous iteration. This alternation between clustering and feature selection continues until there is no change in the set of genes (and partition) between two iterations. The final gene set is removed, and the process repeated on the remaining genes to find other partitions. The algorithm generates a set of binary partitions, along with corresponding sets of genes which differentiate the clusters present in these partitions.

Similar approaches have been used in other algorithms. Ben-Dor *et al *[8] use simulated annealing to efficiently search the space of all binary sample partitions. Xing and Karp [9] use a Normalized Cut algorithm to restrict the search to only the promising partitions and use a similar method of iteration between clustering and feature selection. Von Heydebreck *et al *[10] and Tang *et al *[11] present algorithms that select sample partitions and corresponding gene sets by defining a measure of partition quality and then using greedy search (in the former) and simulated annealing (in the latter) to maximize this measure. Iteration between cluster analysis and gene selection is also used in the "gene shaving" algorithm of Hastie *et al *[12]; though their goal was clustering of genes rather than samples.

## Algorithm

We use a Minimal Spanning Tree (MST) based algorithm [13,14] for clustering along with the Fukuyama-Sugeno clustering measure. Gene selection is done on the basis of the two-sample t-statistic with pooled variance. In the next three subsections we will look in detail at the clustering and feature selection aspects before presenting the formal algorithm.

### Minimal spanning trees

Let *V *= {*x*_1_, *x*_2_..., *x*_*N*_} be a set of points with distances *d*_*ij *_= *d*(*x*_*i*_,*x*_*j*_) defined between all *x*_*i *_and *x*_*j*_. A tree on *V *is a graph with no loops whose vertices are elements of *V *and edge lengths are *d*_*ij*_. A *minimal spanning tree *(MST) is a tree that connects all points such that the sum of the length of the edges is a minimum. An MST can be efficiently computed in O(N^2^) time (including distance calculations) using either Prim's [13] or Kruskal's [14] algorithm.

Deletion of any edge from an MST results in two disconnected trees. Assuming the length of the deleted edge to be *δ *and denoting the sets of nodes in the two trees as *V*_1 _and *V*_2_, we have the property that there are no pairs of points (*x*_1_,*x*_2_), *x*_1 _∈ *V*_1_, *x*_2_∈ *V*_2_such that *d*(*x*_*i*_,*x*_*j*_) <*δ*. Define the smallest distance between any two points, one in *V*_1 _and the other in *V*_2_, as the *separation *between *V*_1 _and *V*_2_. Then we have the result that the separation is at-least *δ*.

The significance of this result is that by deleting an edge of length *δ *we are assured of a partition where the two clusters have a separation of at-least *δ*. This means that if we are interested in looking at all binary partitions with large separations between the clusters, it is sufficient to look at partitions obtained by deleting edges of the MST. Instead of looking at all possible binary partitions (which number 2^*N*-1^-1) our algorithm looks only at partitions obtained by deleting single edges from the MST (which number *N*-1).

Minimal Spanning Trees were initially proposed for clustering by Zahn [15]. More recently, Xu *et al *have used MST for clustering gene expression data [16].

### Clustering measure

To compare the partitions obtained by deleting different edges of the MST, we use the Fukuyama-Sugeno clustering measure [17]. Given a partition *S*_1_, *S*_2 _of the sample index set *S*, with each *S*_*k *_containing *N*_*k *_samples, denote by *μ*_*k *_the mean of the samples in *S*_*k *_and *μ *the global mean of all samples. Also denote by 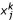 the *j*-th sample in cluster *S*_*k*_. Then the Fukuyama-Sugeno (F-S) clustering measure is defined as


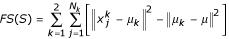


Small values of *FS(S) *are indicative of tight clusters with a large separation between clusters.

We have considered various other clustering measures. The ideal clustering measure should show local minima at each viable partition and have good performance even with a large number of noisy features. We have found the Fukuyama-Sugeno (F-S) measure to give the best performance in these two respects (Supplementary data – Additional file 1).

### Feature selection

For a given partition with two clusters, we can ask if a particular gene shows sufficient differential expression between samples belonging to the different clusters. A gene which is very differently expressed in samples belonging to different clusters can be said to be relevant to the partition or to support the partition. There can be many ways of measuring a gene's support for a partition. Here we use the two sample t-statistic with pooled variance. The t-statistic is computed for each gene to compare the mean expression level in the two clusters. Genes with absolute t-statistic greater than a threshold *T*_*thresh *_are selected. The percentile threshold parameter *P*_*thresh*_*∈*(0,100) is used to compute *T*_*thresh*_. *T*_*thresh *_is the *P*_*thresh*_/2-th percentile of a random variable distributed according to Student's t-distribution with mean zero and *N*-2 degrees of freedom (*N *is the number of samples). Here we use the t-statistic as a heuristic measure of the contribution of each gene to the selected partition; no statistical significance is implied.

The condition for selection of a gene becomes stricter with each iteration. In the first iteration we choose genes with absolute t-statistic greater than *T*_*thresh*_/2. This cutoff increases linearly with the number of iterations until it reaches *T*_*thresh*_. This is done so that we do not lose any useful genes by putting a too-stringent selection criterion before the partition has evolved close to its final form.

### The algorithm

Initially, an MST is created using all the genes; then each binary partition obtained by deleting an edge from the tree is considered as a putative partition. The partition with the minimum value of the F-S clustering measure is selected. The t-statistic is used to select a subset of genes that discriminate between the clusters in this partition. In the next iteration, clustering is done in this set of selected genes. This process continues until the selected gene subset converges (remains the same between two iterations), resulting in a set of genes and the final partition. Having identified a partition and the associated set of genes, these selected genes are removed from the pool of genes. This prevents the algorithm from detecting the same partition the next time. The whole process repeats in the pool of remaining genes to find other partitions.

The inputs to the algorithm are the gene expression matrix {*x*_*s*,*g*_}, the maximum number of partitions to be found *MaxN*_*p *_and percentile threshold *P*_*thresh*_. *P*_*thresh *_is used to compute *T*_*thresh*_. The outer loop of the algorithm runs as long as the number of discovered partitions is less than *MaxN*_*p*_. The set of selected genes *F *is initialized to be the set of all genes *Fset *and the cutoff *t *is initialized as *T*_*thresh *_/2. In the inner loop, an MST is created using the genes in *F*, and for all partitions obtained by deleting single edges from this MST, the F-S measure is calculated. For the partition P* with the lowest F-S measure, genes are selected from *F *based on the t-statistic. These selected genes form the new gene set *F*_*new*_. If *F*_*new *_≠ *F*, the cutoff *t *is increased and another iteration of the inner loop is performed. If *F*_*new *_= *F*, this means that the gene set has remained unchanged between two iterations and the current partition P* along with the current gene set *F *is output. The number of discovered partitions is increased and another iteration of the outer loop is performed.

Since this is an unsupervised method, the partitions picked might be indicative of biological differences that are relevant, irrelevant (like age or sex of patients) or unknown. We control the detection of chance partitions (*i.e. *generated due to noise and not due to any biological difference) by requiring a minimum of 2*M *(1 - *P*_*thresh*_/100) genes in support of a partition (*M *is the total number of genes); the algorithm is terminated if there are fewer.

*P*_*thresh *_plays an important part in the kind of partitions that are extracted. A value of *P*_*thresh *_close to 100 will preferentially extract partitions that are supported by genes with large differential expression between the two clusters. A smaller value of *P*_*thresh *_will pick up partitions that are supported by larger number of genes with lower differential expression between the clusters.

*P*_*thresh *_cannot be interpreted as a measure of the statistical significance of the partitioning since we are doing both the partitioning and the feature selection on the same set of samples. Here we only use *P*_*thresh *_as a parameter for selecting genes.

**Algorithm 1: **Algorithm for iterative clustering

**Input ***MaxN*_*p*_, *P *_*thresh*_, *x*_*s*,*g*_;

*Fset *← {1, 2..., n};

*N*_*p *_← 0; /*Number of currently discovered partitions*/

**Compute ***T *_*thresh*_;

**While ***N*_*p *_<*MaxN*_*p *_**do**

*F *← *Fset*;

*T *← *T *_*thresh*_/2;

While 1 do

**If **length of *F *< 2 *M*(1 - *P*_*thresh*_/100) **then**

/*Not enough genes support partitions*/

exit;

end

Create MST in feature set *F *with metric *d*;

Delete edges one at a time and calculate F-S measure for each ensuring binary partition;

Find partition P* with the lowest F-S measure;

Compute t-statistic *t*_*g *_for all genes g ∈ F for this partition;

Set *F*_*new *_to the set of genes {g**: **|*t*_*g*_| >*t*};

**If ***F*_*new *_= *F ***AND ***t *= *T*_*thresh *_**then**

/*Feature set has converged */

output P* and *F*;

/*Remove genes in *F *from Fset*/

*Fset *← *Fset *\ *F*;

*N*_*p *_= *N*_*p *_+ 1;

break;

else

*F *← *F*_*new*_;

Increase *t*;

end

end

## Results

### Synthetic data

We first tested the algorithm on synthetic data to compare its performance against a hierarchical clustering method at detecting planted partitions. We also estimated the probability of detection of spurious partitions created by noise (*i.e. *the false detection rate).

For both iterative clustering and hierarchical clustering, we found that the probability of detecting the true partition depended only on the Euclidean distance between the clusters in the partition, and for a fixed distance, is relatively insensitive to the number of signal genes (Supplementary data – Additional file 2).

**Figure 2:**
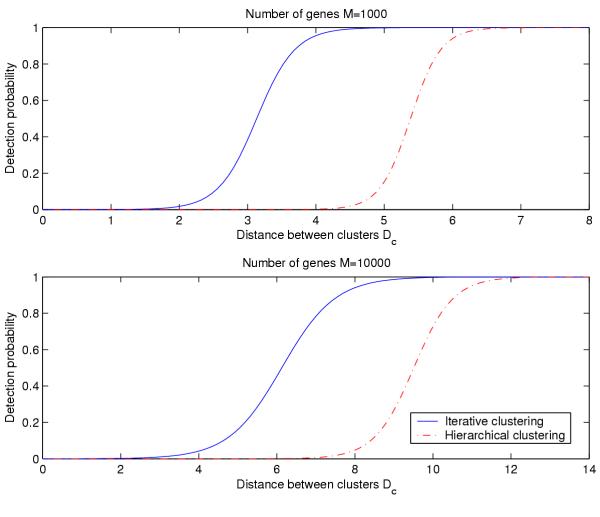
**Detection probability vs. cluster separation. **Probability of detection of the planted partition as a function of the distance between the clusters in the partition.

Figure 2 shows the results of a logistic regression analysis of the dependence of probability of detection of the true partition on the distance between the clusters for both clustering methods. Independent of the total number of genes *N*, iterative clustering detects the planted partition when the two clusters are separated by about half the distance compared to hierarchical clustering. For genes with similar levels of differential expression, this means that the iterative clustering method will detect clusters supported by a quarter of the number of genes required for detection by hierarchical clustering.

The false detection rate was found to be very low: 0.012 for the correlation and 0.011 for the Euclidean distance.

### Microarray data

To test whether classes with strong biological significance can be discovered without knowledge of the class labels, we tested the algorithm on three publicly available sets of micro-array data.

1. BRCA mutation data reported by Hedenfalk *et al *[7] with 6512 cDNA clones of 5361 genes for 7 samples with BRCA1 mutation, 8 samples with BRCA2 mutation and 7 with sporadic breast cancer.

2. Leukemia data-set reported by Golub et al. [6]. Expressions for 7070 genes are provided for 47 acute lymphoblastic leukemia (ALL) samples and 25 acute myeloid leukemia (AML) samples.

3. Lymphoma data-set reported by Alizadeh et al. [1] containing 46 samples of tissues with diffuse large B-cell lymphoma (DLBCL). Expressions for 4026 genes were measured for each of these samples.

It must be noted that if class labels are already available and the goal is to discover genes that differentiate between samples of different classes, then class comparison and class prediction methods exist that are more suitable [21]. Such methods make use of the prior information (in the form of class labels) to detect genes that are significantly differentially expressed between the various classes. The expression of these genes can be used to develop classifiers that predict the class of new samples.

Our iterative method is for cases where no a-priori class labels are assigned. Nevertheless, we have used data for which class labels are known so that there is a ground truth to which the results of the iterative method can be compared. This is similar to what has been done by other authors for validating the results of unsupervised clustering algorithms [8-11].

A-priori gene filtering and normalization performed were similar to that done for the dataset by the original authors. The iterative algorithm was then run with maximum number of partitions *N*_*p *_= 10 and *P*_*thresh *_= 0.999.

**Figure 3:**
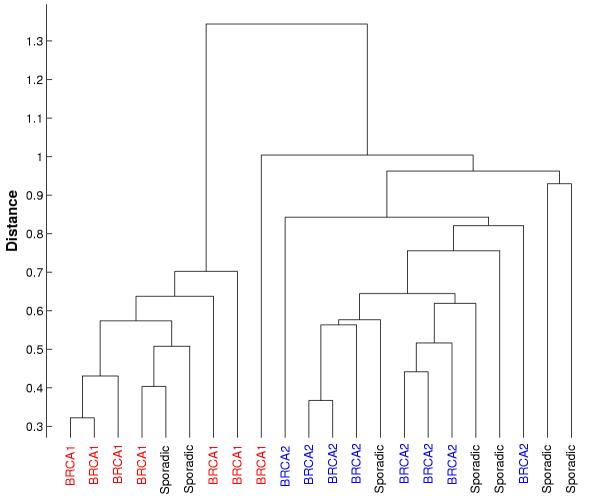
**Hierarchical clustering of BRCA data using selected genes. **Hierarchical clustering of BRCA data using only the genes supporting Partition 4. BRCA1 and BRCA2 are separated with one misclassification.

Table 1 shows the distribution of BRCA1 and BRCA2 samples present in the two clusters for the first four partitions discovered in the BRCA dataset. The fourth partition obtained from the BRCA data separates samples with BRCA1 and BRCA2 mutations with one misclassification. Figure 1 shows the result of hierarchical clustering on the BRCA data-set. The tree structured clustering using all the genes fails to differentiate between samples with BRCA1 and BRCA2 mutations. Figure 3 shows hierarchical clustering using only the genes selected by the iterative clustering method (61 genes). BRCA1 and BRCA2 samples are separated into different branches of the tree with only one misclassification.

With the Leukemia data-set, the first partition obtained matches well with ALL-AML classification, with one cluster containing 46 ALL samples (out of 47 total) and 1 AML sample while the second cluster contains 24 AML samples (out of 25 total) and 1 ALL sample (Table 2).

To see whether the gene set obtained in support of the partition correlating with the AML/ALL classification truly separates ALL and AML samples, we used a split-sample method. The iterative algorithm was used on part of the dataset (38 samples, corresponding to the "training set" used in [6]) and several partitions were obtained. We did not obtain exactly the same partitions as when the whole dataset was used, but the second partition corresponded well to the ALL/AML classification. It contained one cluster with 25 ALL and no AML samples and another cluster with 11 AML and 2 ALL samples. There were 252 genes that were selected in support for this partition.

If the 252 selected genes were truly discriminatory between the ALL and AML samples, then we should be able to separate the two classes in unknown data by unsupervised clustering using these genes. This was verified by clustering the rest of the samples in the dataset (containing 34 samples corresponding to the "testing set" used in [6]) using these genes. Since the iterative algorithm uses a combination of MST and F-S measure to do clustering, we performed the validation using a similar clustering method. An MST was created using the 252 genes and then the edge to be deleted selected according to minimum F-S measure. This identical to the clustering method used in the inner loop of the iterative clustering algorithm (Algorithm 1).

We obtained two clusters with the first cluster containing 20 ALL and 1 AML samples while the second cluster contained 13 AML and no ALL samples. This almost-complete separation of ALL and AML in the testing data shows that the genes selected by the iterative clustering are truly supportive of the partition discovered in the training data.

The biological differences present in the Lymphoma data-set were originally detected using hierarchical clustering [1] after manual selection of genes. We have included our results using the iterative method to show how successful the iterative clustering algorithm is in picking out these disease subclasses (Table 3). The third partition best corresponds to the subclasses discovered by Alizadeh et al. One cluster has 24 GC B-like DLBCL samples and 7 Activated B-like DLBCL samples while the other cluster has 16 Activated B-like DLBCL samples.

The results from the iterative clustering algorithm is compared to that obtained by Overabundance Analysis (OA) [8] (Table 4) and CLIFF [9] (Table 5). Ben-Dor *et al *use the *Jaccard index *[20] to measure the similarity of the partitions discovered by OA to the true biological classes. For comparison, we calculated the same index for partitions discovered by iterative clustering. The Jaccard index ranges from 0 for complete mismatch to 1 for complete match.

Both OA and iterative clustering pick out partitions corresponding to the ALL/AML classification, though OA detects it as the fourth partition while iterative clustering detects it as the first partition. There is a small but definite improvement in the Jaccard index for the results obtained for the Lymphoma data by iterative clustering as compared to OA.

Compared to CLIFF, iterative clustering picks a partition in the Leukemia data that is marginally better (2 misclassified as compared to 3 for CLIFF).

## Discussion

We have presented a clustering method that uses a minimal spanning tree to lead the search for partitions of samples that form good clusters. Iteration between minimal spanning tree cluster analysis and feature selection is used to converge onto partitions that form well separated clusters and gene subsets that support these partitions.

At the convergence of each set of iterations, the result is a partition of the samples and a set of genes that support them. These genes are removed from the pool of genes before searching for other partitions. This removes genes that obscure other partitions supported by smaller numbers of less differentially expressed genes. Genes that support more than one partition will be selected in favor of the partition for which their support is stronger.

Testing on synthetic data shows that the algorithm picks out planted clusters with high accuracy and low false positive rate. Application of the algorithm to breast cancer, leukemia and lymphoma data returns partitions with very well separated clusters, some of which have a strong biological significance. The results are comparable to those obtained by other similar algorithms, and superior to those obtained by standard hierarchical clustering.

The kind of partitions discovered depends very much on the value of *P*_*thresh*_. Values of *P*_*thresh *_close to 100 will give preference to partitions that are supported by a small number of very highly differentially expressed genes. On the other hand, smaller values of *P*_*thresh *_will preferentially detect partitions that are supported by a large number of genes differentially expressed to a lesser degree. If the first application of the algorithm returns several partitions that are correlated with each other, then we could suspect that there is one partition that is supported by a large number of genes and run the algorithm again with a smaller value of *P*_*thresh *_to detect all these genes. We have not been able to specify a single value of *P*_*thresh *_that works in all cases, although the range of values we used (*P*_*thresh *_= 99.9-99.95) works well in most situations.

The partitions and supporting gene sets detected by the use of this algorithm must be further analyzed using gene annotation and clinical data to determine whether they are biologically relevant and worth further investigation. The significance of the detected partitions must be further investigated by evaluating the clinical correlates of patients in different clusters. Clinical observations made on patients, like survival duration, response to therapy and grade of tumor can be compared among the clusters obtained to see if there are any detected partitions whose clusters are correlated with clinical features.

Another, complementary, approach is to analyze the genes that are differentially expressed between two clusters for regulatory relationships with each other or prior known influence on the disease in question. Software tools for searching gene annotations [18], and exploring PubMed and GeneCards for prior published relationships among given genes [19] are available.

The results of these two approaches can help the biologist to formulate hypotheses about the significance of the partitions as well as the role of the selected genes in influencing the course of the disease. Examples of this process can be seen in [1] and [2].

## Methods

Synthetic data were created by generating normally distributed expression profiles for each gene. Each planted partition is supported by a fraction of the genes (called signal genes) which were differentially expressed between the two clusters. Each signal gene is differentially expressed to the same extent. The expressions were normally distributed; *x*_*s*,*g*~_*N *(0,0.5^2^) for samples s belonging to cluster 1 and *x*_*s*,*g*~_*N *(*c*,0.5^2^) for samples belonging to cluster 2. The rest of the genes are not differentially expressed and are called noise genes and are distributed according to a normal distribution *N *(0,0.5^2^). If we have *k *signal genes, each differentially expressed by *c *between the two clusters, the Euclidean distance between the cluster-means will be 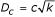.

Sets of synthetic data were generated for number of genes *M *= 1000 and *M *= 10000 with varying fraction of signal genes *ε *and distance between cluster means *D*_*c*_. Each set of data was analyzed both by the iterative and the hierarchical clustering method (using average linkage). The iterative clustering method was used to obtain the first partition discovered using the Euclidean distance (*P*_*thresh *_= 99.95). Hierarchical clustering was used to obtain a tree, and the branch at the highest level was split to produce a partition.

The match of these two partitions with the true partition was calculated and the detection accuracy was assigned 1 if the match was greater than 75% and 0 otherwise. A logistic regression analysis was used to model the dependence of the probability of detection on the distance between the clusters *D*_*c*_.

To estimate the false detection rate, the algorithm was run on synthetic data containing 10 – 100 samples with *P*_*thresh *_= 99.9. Each sample is a 1000-dimensional vector drawn from a multivariate normal distribution. Thus any clusters detected can be expected to be spurious clusters formed by chance.

For the micro-array data, the iterative method was used to detect the first 10 partitions, (*P*_*thresh *_= 99.9) using (1-correlation coefficient) as the distance measure for the MST. For the BRCA data, we also clustered the data using standard hierarchical clustering using centered correlation as the distance metric [4] to compare the results of our algorithm with that obtained by clustering with respect to all genes.

## Supplementary Material

Additional File 1 **Comparison of clustering measures **Synthetic data was created with 100 samples and 1000 genes containing clusters embedded in the first 50 genes. The other 950 genes were normally distributed noise. There are three clusters in the first 50 genes: Samples 1 through 20, samples 21 through 70 and samples 71 through 100. For each binary partition of the points *S*_1 _= {1, 2..., i}, *S*_2 _= {i+1, i+2..., 100}, we calculated the clustering measure. The figure shows the value of the measure for each split point. It can be seen that the Average Linkage and Xie-Beni [22] measures have weak minima and they suffer from extreme values for unbalanced splits. The Log-Likelihood measure has performance similar to the F-S measure but has extreme values for unbalanced splits. Click here for file

Additional File 2 **Detection of true partition for different data parameters **Sets of synthetic data were generated for 1000 and 10000 total number of genes with varying fraction of signal genes *ε *and distance between cluster means *D*_*c*_. The figure shows detection of planted partition for various values of *ε *and *D*_*c*_. Blue points are data for which the percentage match between the first discovered partition and the planted partition is less than 75%. The red points are data for which the match is greater than 75%. Detection (match > 75%) depends only on the distance between the clusters for both hierarchical and iterative clustering. Click here for file

## Figures and Tables

**Table 1:** Results on BRCA data-set

**Partition number**	**Cluster number**	**BRCA1**	**BRCA2**	**Number of genes selected**
**1**	1	0	4	80
	2	7	4	
**2**	1	7	4	110
	2	0	4	
**3**	1	5	3	73
	2	2	5	
**4**	1	6	0	61
	2	1	8	

**Table 2:** Results on Leukemia data-set

**Partition number**	**Cluster number**	**AML**	**ALL**	**Number of genes selected**
**1**	1	1	46	578
	2	24	1	
**2**	1	4	29	650
	2	21	18	
**3**	1	25	38	108
	2	0	9	
**4**	1	20	27	81
	2	5	20	

**Table 3:** Results on Lymphoma data-set

**Partition number**	**Cluster number**	**GC B-like DLBCL**	**Activated B-like DLBCL**	**Number of genes selected**
**1**	1	20	22	121
	2	4	1	
**2**	1	3	4	226
	2	21	19	
**3**	1	24	7	156
	2	0	16	
**4**	1	15	12	309
	2	9	11	

**Table 4:** Comparison of results with that obtained using Overabundance Analysis (Ben-Dor et al [8])

**Data-set**	Jaccard index of first 4 partitions discovered by iterative clustering	Jaccard index of first 4 partitions discovered by Overabundance Analysis
**Leukemia**	0.906	0.469
	0.424	0.344
	0.454	0.469
	0.378	0.949
**Lymphoma DLBCL**	0.452	0.362
	0.429	0.324
	0.611	0.354
	0.343	0.350

**Table 5:** Comparison of results with that obtained using CLIFF (Xing and Karp [9])

	**Cluster number**	**AML**	**ALL**
**Iterative clustering**	1	1	46
	2	24	1
**CLIFF**	1	0	44
	2	25	3
